# Application of artificial muscle e-rubber for healthcare sensing: verification of measurement properties as a smart insole

**DOI:** 10.3389/fbioe.2025.1639630

**Published:** 2025-08-21

**Authors:** Hidemasa Yoneda, Takashi Yamaga, Takeshi Fujiwara, Yoko Komori, Masatoshi Shimada, Yuki Kato, Shintaro Oyama, Shingo Shimoda, Michiro Yamamoto, Hitoshi Hirata

**Affiliations:** ^1^ Human Enhancement and Hand Surgery, Nagoya University, Nagoya, Japan; ^2^ Orthopedic Surgery, Aichi Medical University, Nagakute, Japan; ^3^ Orthopedic Surgery, Tsushima City Hospital, Tsushima, Japan; ^4^ New Value Business, Toyoda Gosei Co., Ltd., Ama, Japan; ^5^ Orthopedic Surgery, Chunichi Hospital, Nagoya, Japan; ^6^ Graduate School of Medicine, Nagoya University, Nagoya, Japan

**Keywords:** e-rubber, artificial muscle, capacitance sensor, dielectric elastomer, smart insole, sensing for healthcare

## Abstract

Electroactive polymer (EAP) artificial muscles are gaining attention in robotic control technologies. Among them, the development of self-sensing actuators that integrate sensing mechanisms within artificial muscles is highly anticipated. This study aimed to evaluate the accuracy and precision of the sensing capabilities of the e-Rubber (eR), an artificial muscle developed by Toyoda Gosei Co., Ltd., and to investigate its potential for healthcare sensing applications such as smart insoles. The objective was to transform the eR into a thin capacitor and estimate the applied load by sensing minute changes in the capacitance. The changes in the EAP dielectric constant, electrode area, and inter-electrode distance, all of which define the capacitance, are non-linear functions. The relationship with the external force also exhibits nonlinearity. To address this issue, we experimentally plotted the load and capacitance changes and derived a regression equation. We evaluated the sensing characteristics of both a stand-alone sensor and a sensor embedded in a smart insole, followed by a precision verification of the load estimation using the derived regression equation. Load–capacitance changes were measured up to 400 N at three conditions: 23 °C and 50% humidity, 40 °C and 50% humidity, and 40 °C and 80% humidity. For the standalone sensor, the coefficient of variation was less than 1.25% and the confidence interval was 0.25%, indicating high precision. However, for the sensor embedded within the insole housing, the coefficient of variation increased to less than 8%, and the confidence interval was 1.5%, likely owing to the influence of gaps within the insole structure. Regarding the load estimation equation, a 5th-order polynomial approximation (R^2^ >0.999) demonstrated the best fit, indicating that it is sufficiently accurate for healthcare sensing applications. Although capacitance-based sensors are increasingly being used in biomedical monitoring for pressure and load measurements owing to their durability and high sensitivity, their primary challenge lies in the nonlinearity of the sensing results. Although this challenge also exists for capacitance sensors utilizing artificial muscles, our study shows that developing a regression equation based on the experimental relationship between the load and capacitance changes can yield sufficient precision for practical healthcare applications.

## 1 Introduction

Artificial muscles that exhibit expansion, contraction, and other movements, just like biological muscles, have attracted significant attention in robotics control (Jing et al., 2023). Typical artificial muscles driven by electrical energy utilize electroactive polymers (EAPs), which are polymeric materials capable of changing their shapes and dimensions, sandwiched between electrodes. EAPs include dielectric elastomers, piezoelectric polymers, and adsorption films. When a voltage is applied to the electrodes, the EAP deforms, resulting in actuation (Maksimkin et al., 2022).

The e-rubber (eR) developed by Toyoda Gosei Co., Ltd. is an EAP-type artificial muscle that employs a dielectric elastomer. The eR is composed of multiple layers, including the EAP and electrodes. When current flows, the dielectric elastomer layer deforms and is converted into an actuator movement (video). To maintain elasticity while ensuring lightness, a dielectric elastomer was initially developed using slide-ring materials (Sapsford and Michieletto, 2025).

In the healthcare field, early intervention is crucial for preventing the progression of many conditions to severe stages. For example, osteoarthritis (OA) of the knee can be definitively diagnosed with radiography, but there are no established biomarkers for its detection in the early stages, except magnetic resonance imaging (MRI) (Li et al., 2023; Piccolo et al., 2023). However, it is known that gait abnormalities are present in the early phases of the disease (Duffell et al., 2014). Consequently, gait analysis may enable the detection of early pathological changes even before they are visible on radiographs (Wipperman et al., 2024).

In this context, smart insoles equipped with soft sensors have been widely reported, and the technology itself is not novel. Our goal, however, is to develop a smart insole specifically capable of early detection of degenerative diseases such as OA. To contribute effectively to preventive medicine in healthcare, we set two primary objectives: achieving a consumer-friendly price point of under $250 for widespread adoption and enabling preventive interventions using the insole. This contrasts sharply with high-end, high-precision systems such as Moticon OpenGo (Munich, Germany), Novel Pedar (Munich, Germany), and Tekscan F-Scan (Norwood, MA, United States), which are priced over $2,000, with some exceeding $10,000, making them prohibitive for widespread preventive use.

In addition to their role as actuators, artificial muscles can be utilized as sensors capable of detecting minute forces. This is achieved by treating the artificial muscle as a capacitor and leveraging the change in its capacitance owing to external forces (Jung et al., 2008). A recent trend in artificial muscle research is the development of “self-sensing actuators,” which combine actuation functionality with the ability to sense their activity. This approach mimics the spindles found in human muscles, enabling self-sensing of shape changes in the artificial muscle to control the actuator. This is expected to be applied to robot control using artificial intelligence (Gonzalez-Vazquez et al., 2023). By integrating a thin sensing actuator into a smart insole, we envision the possibility of not only sensing but also delivering interventions based on the sensor data. For instance, it could alert users to a high risk of falling by sending signals from the sole or enhance plantar sensation for patients with diabetes or peripheral neuropathy (Ahmad et al., 2024). Such plantar actuation could potentially promote active rehabilitation.

**FIGURE 1:**
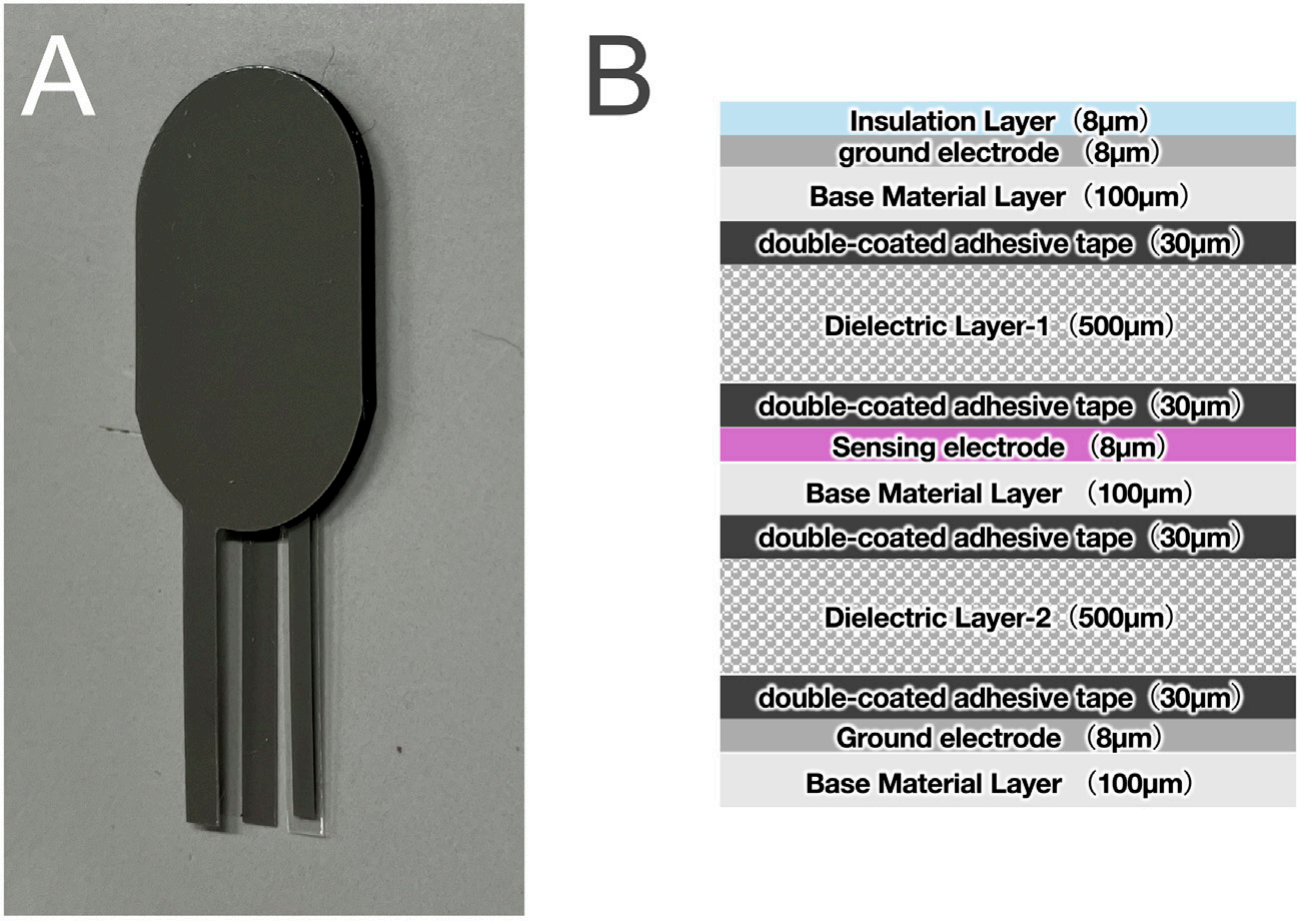
**(A)** An eR sensor created by making artificial muscle thinner, and **(B)** its cross-sectional view.

Therefore, we chose eR for our smart insole development, and we modified the eR into a thin sensor to monitor dynamic plantar pressure (Figure 1). The sensor was designed with a sandwich-like structure to mitigate capacitive noise from contact with the plantar skin or socks. Furthermore, a combination of two distinct materials was utilized to create a design that reduces hysteresis. To realize eR smart insole, several challenges must be addressed, including ensuring the accuracy and reliability of the sensing component, processing measurement data in real-time, and delivering appropriate actuation based on the results. As a first step, we must verify the accuracy and precision of the eR sensing capabilities. Because a thorough validation of eR for such precise healthcare applications has not been previously undertaken, this study aimed to perform this foundational evaluation. Consequently, this study aimed to evaluate the measurement accuracy of the sensor with the premise of its application as a plantar pressure sensor.

## 2 Materials and methods

### 2.1 Structure of the eR sensor

The eR sensor is composed of two 0.5 mm thick urethane foam dielectric layers and three silver paste electrodes, resulting in a total thickness of 1.47 mm. The dielectric layers possess distinct densities and mechanical properties, a design that expands the measurable load range. Specifically, Dielectric Layer 1 has a density of 150 kg/m^3^, a compressive stress of 0.006 MPa at 25% strain, and a compression set of 1.0%, whereas Dielectric Layer 2 has a density of 240 kg/m^3^, a compressive stress of 0.022 MPa at 25% strain, and a compression set of 4.2%.

This configuration, utilizing dielectrics with different elastic moduli, facilitates stepwise deformation in the thickness direction. Consequently, compared to a single-dielectric sensor, the capacitance change does not saturate, enabling a detectable output over a wider input range (Zhu et al., 2022). Furthermore, the top and bottom electrodes are connected to 0V to serve as ground electrodes. These act as electrostatic shields to mitigate the influence of external noise.

### 2.2 eR sensing theory

The principle of the eR sensor is to estimate the external force by treating the eR as a parallel-plate capacitor and utilizing the change in capacitance. When the eR deforms owing to an external force, the electrode area A and the distance between the electrodes d change; consequently, the capacitance changes. The capacitance C stored in eR is defined by the following Equation 1:

$$C=\frac{\varepsilon_{r}\varepsilon_{0}A}{d}$$ (1)

Where ϵ_*r*_: relative permittivity of the elastomer, ϵ_0_: permittivity of vacuum, A: electrode area, d: distance between electrodes. ϵ_0_ = 8.854 × 10–^12^ F/m.

By measuring the change in capacitance (ΔC) before and after the application of an external force, the external force can be inversely calculated as Equation 2. C was defined by considering changes in each parameter.

$$\Delta C=\frac{\left(\varepsilon_{r0}+\Delta \varepsilon_{r}\right)\varepsilon_{0}\left(A_{0}+\Delta A\right)}{d_{0}+\Delta d}-\frac{\varepsilon_{r0}\varepsilon_{0}A_{0}}{d_{0}}$$ (2)

The relative permittivity of dielectric elastomers is generally unaffected by external forces or changes in shape, with negligible variations observed (Zhao and Suo, 2010). The dielectric layer of e-rubber uses urethane foam, which consists of a bulk material and air bubbles with different relative permittivity (Figure 1). We hypothesize that while the relative permittivity of urethane foam remains largely unchanged at small strains, it changes under load as air bubbles collapse, causing the bulk material’s properties to become dominant (O'Neill et al., 2022).

The change in capacitance is defined by the change in the elastomer permittivity, electrode area, and distance between the electrodes. However, since the changes in Δd and A are not linear but rather non-linear functions, and because the elastomer’s permittivity ϵ_*r*_ changes due to density variations caused by stress and strain, the capacitance becomes a non-linear function.

Next, we examined the relationship between ΔC and the external force F. Assuming that the electrode area of eR and the relative permittivity of the elastomer does not change, the initial capacitance C_0_ and the capacitance C_1_ after loading are given by Equation 1 as Equations 3, 4:

$$C_{0}=\varepsilon_{r}\varepsilon_{0}\frac{A}{d_{0}}$$ (3)

$$C_{1}=\varepsilon_{r}\varepsilon_{0}\frac{A}{d_{0}-\Delta d}$$ (4)

Thus, the change in capacitance ΔC is calculated by Equation 5:

$$\Delta C=C_{1}-C_{0}=\varepsilon_{r}\varepsilon_{0}A\left(\frac{1}{d_{0}-\Delta d}-\frac{1}{d_{0}}\right)$$ (5)

When a normal load F is applied to an elastic dielectric (thickness d_0_, area A, Young’s modulus E), the change in thickness Δd is calculated with Equation 6:

$$\Delta d=\frac{Fd_{0}}{EA}$$ (6)

Substituting (6) into (5), ΔC is calculated with Equation 7

$$\Delta C=\varepsilon_{r}\varepsilon_{0}A\cdot \frac{1}{d_{0}}\left(\frac{1}{1-\frac{F}{EA}}-1\right)=C_{0}\left(\frac{1}{1-\frac{F}{EA}}-1\right)$$ (7)

Therefore, the relationship between ΔC and F can be defined as a non-linear relationship. Furthermore, for small deformations where F≪EA, using the Maclaurin expansion, ΔC is approximated by Equation 8:

$$\Delta C\approx C_{0}\cdot \left(\frac{F}{EA}\right)$$ (8)

This indicates that, in the small-deformation region, where the external force is considerably small, the relationship between the load and capacitance can be defined as linear. However, as the external force increases, the electrode area of the eR, the relative permittivity of the elastomer change, and the structural hysteresis of the elastomer also have an effect. Consequently, the relationship between the change in capacitance and external force applied to eR may deviate from the results obtained using this equation. Therefore, we decided to plot the external force and capacitance changes experimentally and perform regression using curve fitting.

### 2.3 Measurement of load and capacitance change, and evaluation of sensor measurement characteristics from obtained results

**FIGURE 2:**
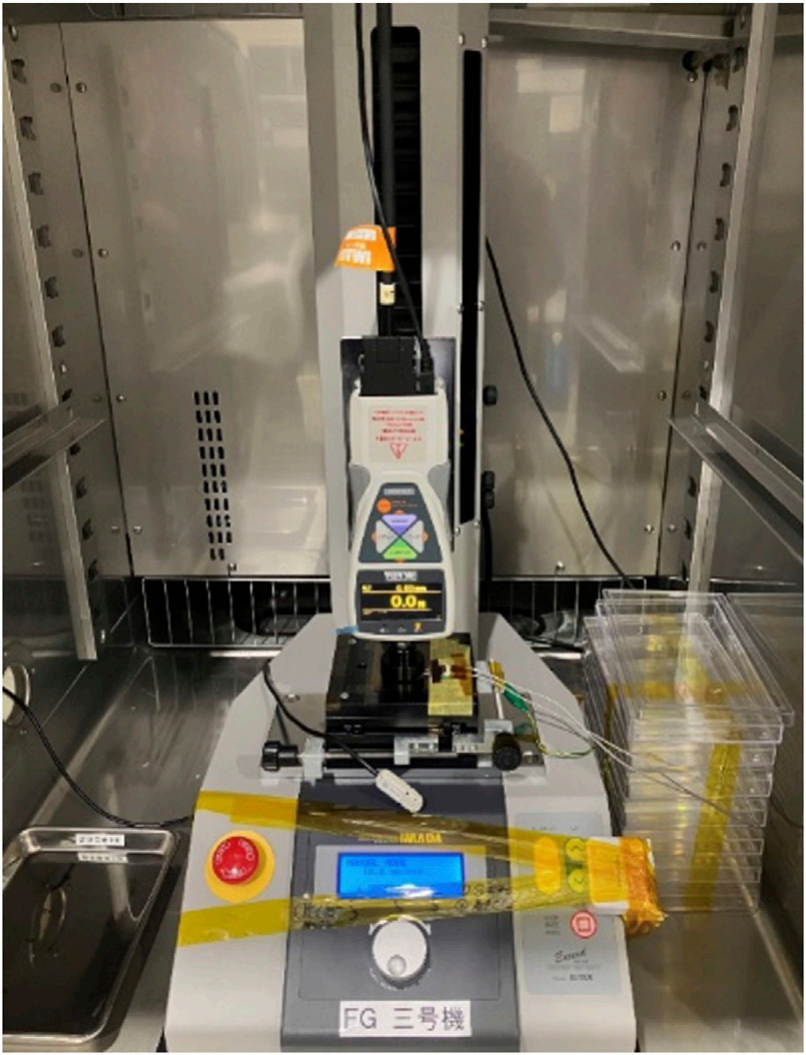
Load application test of the eR sensor using a force gauge installed within a constant temperature chamber. Capacitance was measured using an LCR meter placed externally.

**FIGURE 3:**
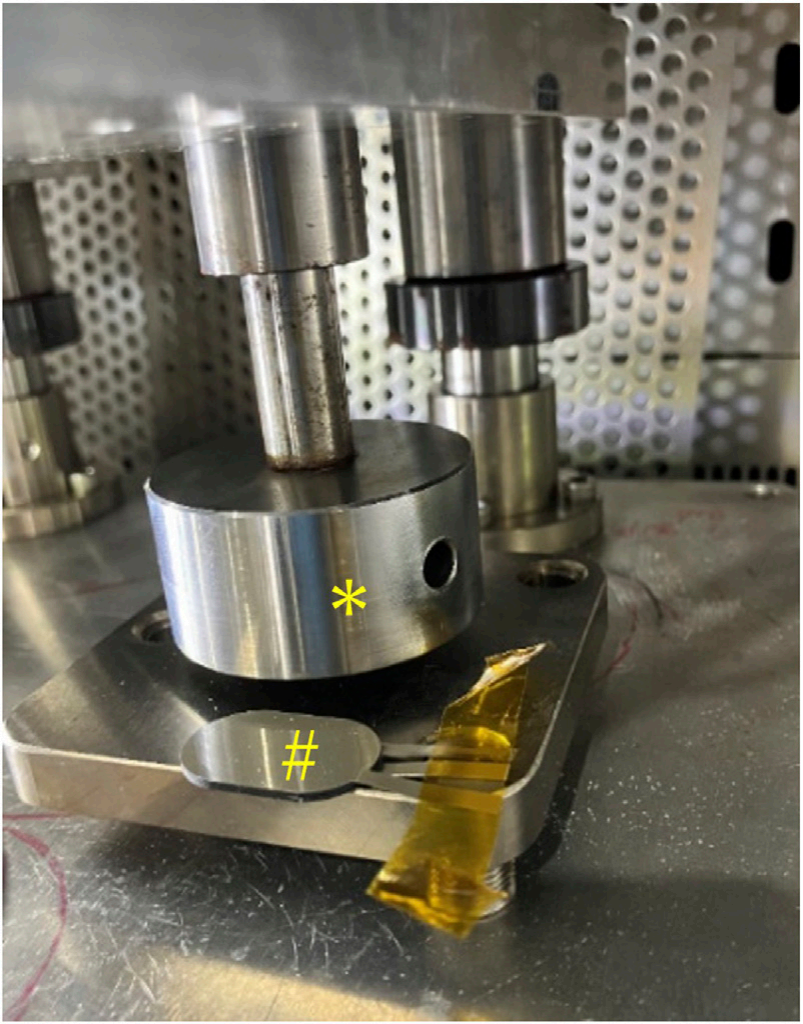
Durability test conducted using a pusher attached to an air cylinder, installed within a constant temperature chamber; *: Pusher, #: eR sensor.

The measurements were conducted inside a TX411N constant-temperature chamber (Kusumoto Chemicals, Ltd., Tokyo, Japan) with a force gauge (EMX-1000N, Imada, Toyohashi, Japan) installed to ensure constant humidity and temperature (Figure 2). A load was continuously applied to the sensor using a force gauge, and the change in capacitance was measured. Loads were applied from 0 to 400 N at a rate of 0.5 mm/s (Figure 3). Capacitance was measured using an LCR meter (IM 3536, Hioki EE Corp., Ueda, Japan) and a custom-developed program.

We used 30 eR samples and performed three measurements on each sample. Since EAP properties vary with temperature and humidity, measurements were taken under three conditions: Condition A (room temperature assumed: 23 °C, 50% humidity), Condition B (close to biological monitoring conditions: 40 °C, 50% humidity), and Condition C (close to conditions for clothes and shoes in a sweating state: 40 °C, 80% humidity).

Polymeric materials such as EAPs exhibit the Mullins effect, where the elasticity changes after repeated loading. In applications such as measuring pressure with insoles during walking or pinching movements of the fingers, repeated loading operations can potentially alter the measurement values. Therefore, as a durability test, we performed frequent pressure operations and measured the changes before and after the operation. The durability test setup included a constant-temperature chamber (SH-642, Espec Corp., Osaka, Japan) equipped with a pusher attached to an air cylinder (COQ2B40-40DZ, SMC, Tokyo, Japan), which was monitored using a load cell (LUR-2KNSA1, KYOWA Electronic Instruments, Chofu, Japan) (Figure 3). For the durability test, simulating actual walking, we applied 200,000 cycles of reciprocal loading from 0 to 100 N per second within a constant-temperature chamber at 40 °C and 80% humidity.

To evaluate the measurement characteristics of the eR sensor, we assessed its reliability and validity based on the Consensus-based Standards for the selection of health status Measurement Instruments (COSMIN) guideline (Mokkink et al., 2010). Reliability refers to the dispersion of sample values and its magnitude represents precision. Conversely, the closeness of the sample mean indicates that the population mean was calculated with accuracy. Because the sample values increase with external force, and consequently, the standard deviation also increases, we calculated them as coefficients of variation. Accuracy is the difference between the sample and population means, calculated using a 95% confidence interval.

To measure the measurement characteristics, we calculated the coefficient of variation and confidence interval for six conditions (Conditions A, B, and C before and after durability testing) based on the measurement values from all samples at load points of 50, 100, 150, 200, 300, and 400 N. We used t-distribution to calculate the 95% confidence interval, defining the confidence interval width as the ratio of the estimated population mean divided by the 95% confidence interval.

### 2.4 Hysteresis of dielectric layers

We investigated the hysteresis of the two dielectric layer materials used as Dielectric layers 1 and 2 (Figure 1) in the eR sensor, using a force gauge according to the procedure described in Section 2.3. The samples were 32 mm in diameter before compression. The tests were conducted at 20 °C and 45% relative humidity. Each sample was compressed from 0 N to 400 N over 12.3 s. We analyzed the relationships between nominal stress (load divided by sample area) and capacitance, and between nominal strain (deformation divided by initial thickness) and capacitance change, during both loading and unloading cycles.

To evaluate hysteresis, we calculated two metrics for each relationship: The first was the mean hysteresis error (%FS), defined as the average of the absolute differences in capacitance between the loading and unloading curves at a given input value, normalized by the full-scale output. The second was the normalized hysteresis loop area, obtained by integrating the area enclosed by the loading and unloading curves using the trapezoidal rule and dividing it by the product of the full-scale input and output ranges. These values provided quantitative measures of the reversibility and repeatability of the sensor response.

### 2.5 Insertion into insoles

**FIGURE 4:**
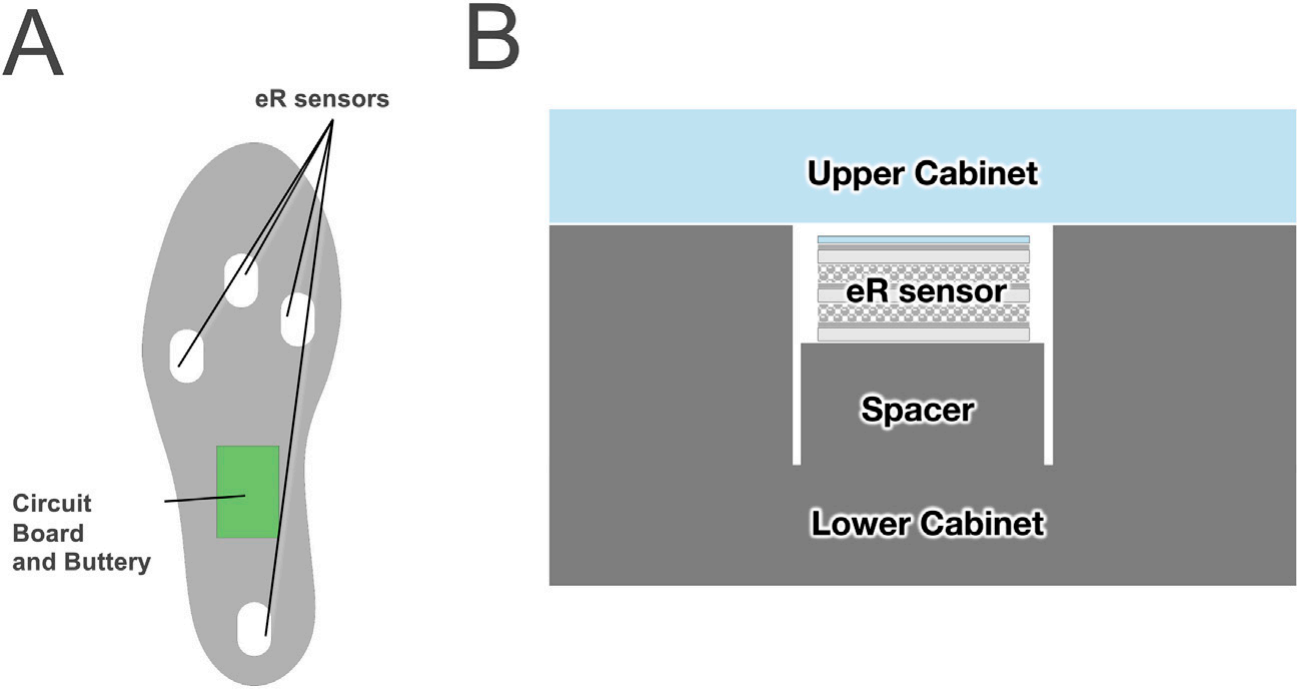
Arrangement of eR sensors within the insole **(A)** and cross-sectional view **(B)**.

We aimed to create a smart insole for measuring plantar pressure using eR and to verify the measurement accuracy of the sensor for this purpose. The planned smart insole consists of four sensors: one at the forefoot, one each on the medial and lateral sides of the midfoot, and one on the hindfoot (Figure 4A). As shown in the figure, the insole cross-sectional feature sensors were sandwiched between the Ethylene Vinyl Acetate (EVA) foam and polyurethane foam (Figure 4B). We created six smart insoles for measurement and performed three measurements for each insole.

### 2.6 Evaluation of load-capacitance change from insoles and measurement characteristics of insole-embedded sensors

**FIGURE 5:**
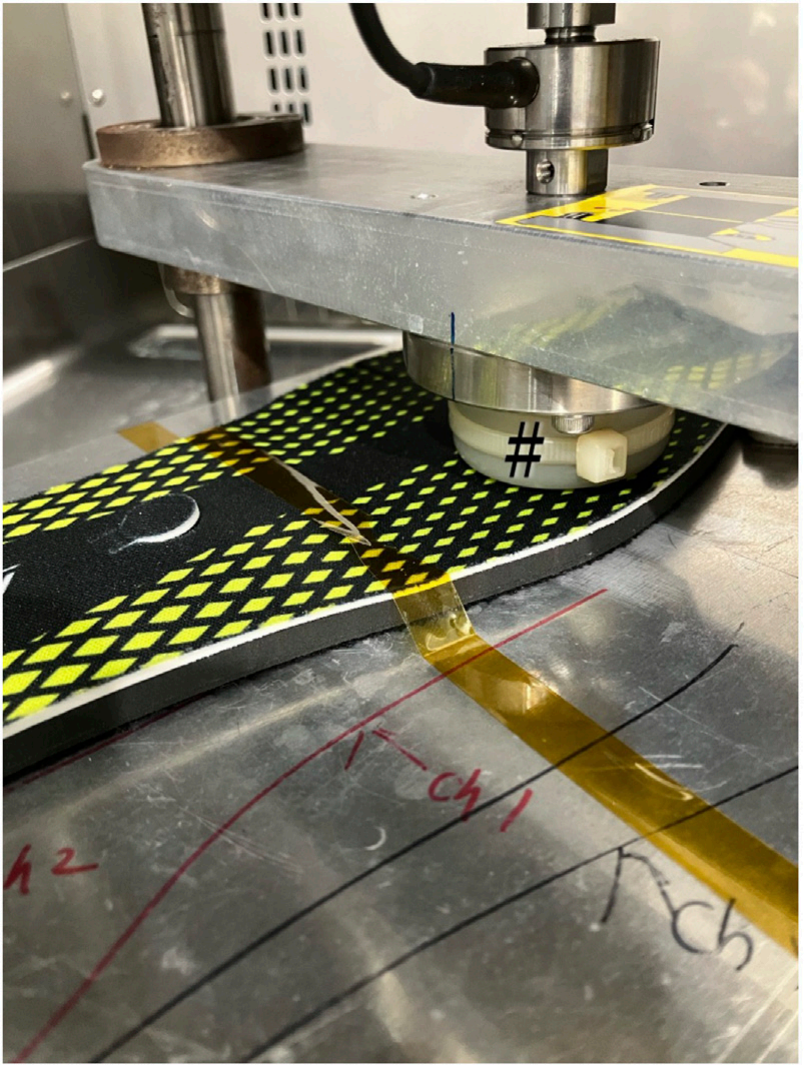
Load application test on the insole installed within a constant temperature chamber. Using a pusher (#), the sensor part is pressed to measure the amount of applied pressure and the change in capacitance.

The measurement characteristics of the sensors inserted into the insoles were evaluated, as described in Section 2.4. The measurement and durability testing equipment were the same as those described in section 2.3. The load application speed was 10 mm/min, with loads ranging from 0 to 400 N. To simulate the plantar pressure measurement, the force gauge indenter was made of POM resin and Si rubber, rather than the metal indenter used in the 2.2’s mold (Figure 5). The indenter was used to apply a load to the insole (Figure 5). For the load test, we similarly changed the indenter and applied 200,000 cycles of reciprocal loading from 0 to 230 N per second in a constant-temperature chamber at 40 °C and 80% humidity.

The 30 eRs were tested under load conditions similar to those described for the sensor in Section 2.3. For each sample, tests were conducted three times, both before and after load application, under Conditions A, B, and C.

### 2.7 Creation of regression equations for load-capacitance in insole-embedded sensors and verification of load estimation accuracy

We plotted the relationship between the obtained ΔC and the applied load, then calculated a polynomial to represent this relationship using curve fitting. The least squares method was used for the calculation, and Python’s NumPy polyfit and poly1d libraries were used for the computation. The candidate equations for curve fitting included a 5th-order polynomial, natural exponential functions, and integer-based exponential functions. Machine learning methods can be used as an alternative to curve fitting. Nevertheless, they were not considered due to the substantial processing burden they would impose on the insole’s circuitry. The accuracy of the curve fitting was evaluated using the coefficient of determination (R2) and the Root Mean Squared Error (RMSE).

### 2.8 Dynamic characteristics analysis of insole-embedded sensors

**FIGURE 6:**
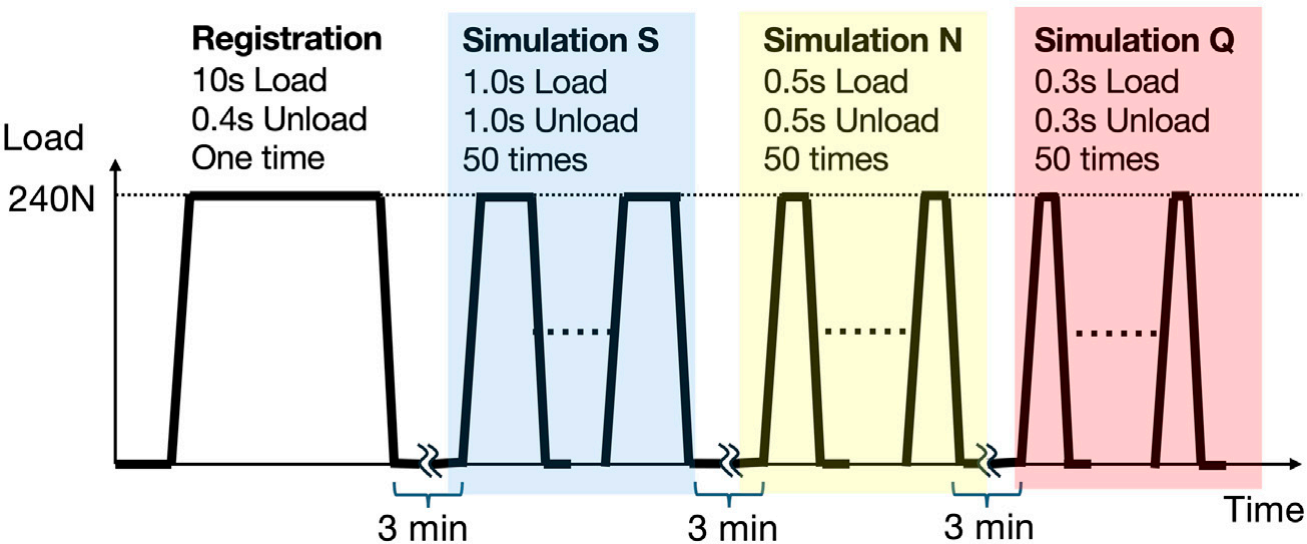
Load application pattern in dynamic characteristics analysis. Initially, a single load is applied for 10 s, followed by a 3-min no-load period. Subsequently, to simulate a slow walk (Simulation S), a 2-s cycle (1-s load, 1-s no-load) is repeated 50 times. Next, to simulate a normal walk (Simulation N), a 1-s cycle (0.5-s load, 0.5-s no-load) is repeated 50 times. Finally, to simulate a quick walk (Simulation Q), a 0.6-s cycle (0.3-s load, 0.3-s no-load) is repeated 50 times. For the analysis of S, N, and Q, the last 40 of the 50 loading cycles are used.

Using the system described in section 2.4, a dynamic characteristics analysis was conducted by applying continuous loads to the insole sensors to simulate walking. The loading cycles were set to simulate different walking speeds: a 2-s cycle for a slow walk (S), a 1-s cycle for a normal walk (N), and a 0.6-s cycle for a quick walk (Q). A load of 0–240 N was repeatedly applied 50 times for each condition (Figure 6). The initial 10 cycles of each loading session were excluded from the data, and the subsequent 40 cycles were used for the dynamic analysis. The capacitance of the insole was sampled every 20 ms. This loading procedure was performed under the three environmental conditions A through C, detailed in section 2.4. The same durability test as in section 2.4 was conducted to examine changes before and after the test. The investigated parameters were the difference between the loading interval and the detected waveform peak interval, and the signal drift rate. To assess whether the two groups were statistically equivalent, we conducted an equivalence test using the two one-sided test (TOST) procedure. The equivalence margin was pre-specified as 50% of the standard deviation. Equivalence was concluded if the 90% confidence interval of the mean difference fell entirely within the predefined margin, and both one-sided p-values were below 0.05. Analyses were performed using Python 3.12. The signal drift rate was calculated for all conditions from section 2. By dividing the difference in estimated load (derived from capacitance) before and after the durability test by the pre-test estimated load.

## 3 Results

### 3.1 Load-capacitance relationship and measurement characteristics of the sensor unit alone

**FIGURE 7:**
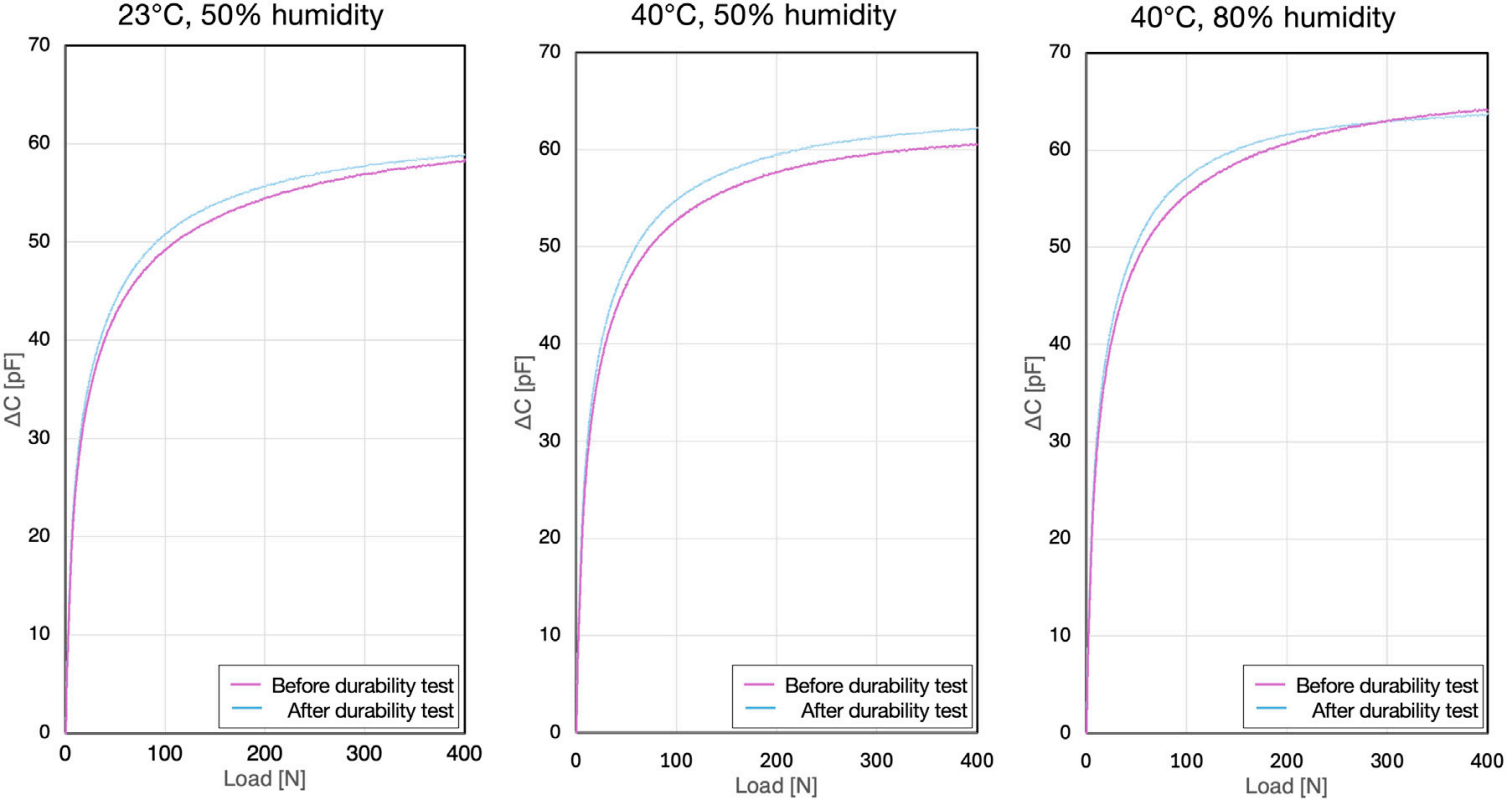
Capacitance-load curves for the standalone sensor.

The relationship between the load value from the continuous pressure applied by the force gauge and the change in capacitance of the sensor unit alone showed a linear increase in the small deformation region up to 20 N under all conditions (A, B, and C). Beyond 20 N, it exhibits a non-linear monotonic increase, closely resembling a logarithmic curve (Figure 7). Although there were differences between individual samples, no variations were observed among the samples. After the durability test, a slight change in the capacitance output was observed; however, the shape of the curve remained consistent (Figure 7).

The coefficients of variation and confidence interval widths for all samples at the specified loads are listed in Tables 1–3. The coefficient of variation was less than 1.25%, indicating that sample variability was within acceptable precision limits. Furthermore, the confidence interval width showed a slight increasing trend after durability testing, but was generally less than 0.25%, which was deemed acceptable in terms of accuracy.

**TABLE 1:** Measurement Characteristics of the Standalone Sensor Unit (23 °C, 50% humidity).

	Before Durability Test			After Durability Test		
Load(N)	Capacitance Mean ± SD (pF)	CV(%)	Width of 95%CI (%)	Capacitance Mean ± SD (pF)	CV(%)	Width of 95%CI (%)
50	42.13 ± 0.45	1.07	0.19	43.77 ± 0.5	1.14	0.18
100	48.74 ± 0.4	0.82	0.18	50.5 ± 0.39	0.77	0.18
150	51.93 ± 0.34	0.65	0.19	53.48 ± 0.36	0.67	0.17
200	53.92 ± 0.29	0.54	0.15	55.25 ± 0.35	0.63	0.18
300	56.18 ± 0.35	0.62	0.21	57.19 ± 0.43	0.75	0.23
400	57.4 ± 0.45	0.78	0.19	58.22 ± 0.52	0.89	0.21

SD, standard deviation; CV, coefficient of variation.

CI, confidence interval.

**TABLE 2:** Measurement Characteristics of the Standalone Sensor Unit (40 °C, 50% humidity).

	Before Durability Test			After Durability Test		
Load(N)	Capacitance Mean ± SD (pF)	CV(%)	Width of 95%CI (%)	Capacitance Mean ± SD (pF)	CV(%)	Width of 95%CI (%)
50	45.63 ± 0.41	0.90	0.15	47.53 ± 0.51	1.07	0.19
100	52.43 ± 0.33	0.63	0.15	54.3 ± 0.36	0.66	0.13
150	55.67 ± 0.27	0.49	0.13	57.24 ± 0.31	0.54	0.12
200	57.65 ± 0.23	0.40	0.12	58.97 ± 0.3	0.51	0.15
300	59.86 ± 0.26	0.43	0.15	60.79 ± 0.35	0.58	0.18
400	61.05 ± 0.33	0.54	0.11	61.69 ± 0.41	0.66	0.15

**TABLE 3:** Measurement Characteristics of the Standalone Sensor Unit (40 °C, 80% humidity).

	Before Durability Test			After Durability Test		
Load(N)	Capacitance Mean ± SD (pF)	CV(%)	Width of 95%CI (%)	Capacitance Mean ± SD (pF)	CV(%)	Width of 95%CI (%)
50	48.03 ± 0.43	0.90	0.17	50.15 ± 0.63	1.26	0.20
100	55 ± 0.35	0.64	0.15	57.06 ± 0.48	0.84	0.18
150	58.28 ± 0.31	0.53	0.14	60.02 ± 0.41	0.68	0.17
200	60.29 ± 0.32	0.53	0.15	61.7 ± 0.41	0.66	0.18
300	62.48 ± 0.4	0.64	0.21	63.47 ± 0.49	0.77	0.22
400	63.59 ± 0.52	0.82	0.19	64.37 ± 0.55	0.85	0.17

### 3.2 Evaluation of load-capacitance change and measurement characteristics of insole-embedded sensors

**FIGURE 8:**
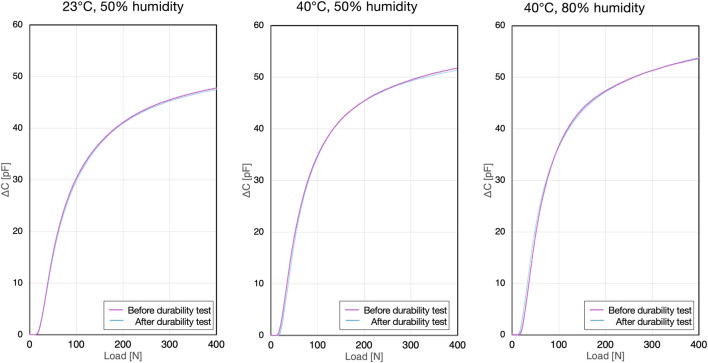
Capacitance-load curves for the sensor within the insole casing.

When comparing measurements from the sensor unit alone to those from sensors inserted into the insole casing, differences in the load-capacitance relationship were observed. Specifically, the sensors within the insole casing showed a gentler increase in the load-capacitance curve compared to the standalone sensor, which was influenced by the gaps within the insole casing (Figure 8).

The coefficients of variation and confidence interval widths at the specified loads are listed in Tables 4–6. The coefficient of variation was less than 8%, indicating that while the sample variability was greater than that of the standalone sensor, it was still considered within acceptable precision limits. The confidence interval width also showed a slight increasing trend after durability testing, but was generally less than 1.5%, which was deemed acceptable in terms of accuracy.

**TABLE 4:** Measurement Characteristics of Insole-Embedded Sensors (23 °C, 50% humidity).

	Before Durability Test			After Durability Test		
Load(N)	Capacitance Mean ± SD (pF)	CV(%)	Width of 95%CI (%)	Capacitance Mean ± SD (pF)	CV(%)	Width of 95%CI (%)
50	45.75 ± 1.78	3.89	0.57	48.37 ± 3.48	7.19	1.14
100	102.75 ± 1.22	1.19	0.21	108.33 ± 4.87	4.50	0.83
150	148.52 ± 1.51	1.02	0.19	158.85 ± 9.61	6.05	1.16
200	199.93 ± 1.64	0.82	0.16	217.77 ± 16.34	7.50	1.42
300	304.33 ± 1.98	0.65	0.15	334.32 ± 27.51	8.23	1.94
400	385.85 ± 2.49	0.65	0.17	423.52 ± 32.45	7.66	2.08

**TABLE 5:** Measurement Characteristics of Insole-Embedded Sensors (40 °C, 50% humidity).

	Before Durability Test			After Durability Test		
Load(N)	Capacitance Mean ± SD (pF)	CV(%)	Width of 95%CI (%)	Capacitance Mean ± SD (pF)	CV(%)	Width of 95%CI (%)
50	39.62 ± 2.72	6.87	1.03	42.57 ± 3.1	7.28	1.13
100	85.61 ± 3.83	4.47	0.77	90.25 ± 4.58	5.07	0.90
150	129.87 ± 6.54	5.04	0.87	141.32 ± 10.53	7.45	1.32
200	179.4 ± 8.97	5.00	0.89	198.44 ± 15.46	7.79	1.39
300	277.68 ± 12.57	4.53	1.06	308.47 ± 23.43	7.60	1.81
400	362.26 ± 13.74	3.79	0.00	397.57 ± 28.13	7.08	1.97

**TABLE 6:** Measurement Characteristics of Insole-Embedded Sensors (40 °C, 80% humidity).

	Before Durability Test			After Durability Test		
Load(N)	Capacitance Mean ± SD (pF)	CV(%)	Width of 95%CI (%)	Capacitance Mean ± SD (pF)	CV(%)	Width of 95%CI (%)
50	43.79 ± 3.45	7.88	1.16	45.84 ± 3.95	8.62	1.33
100	96.34 ± 5.49	5.70	0.94	102.87 ± 4.87	4.73	0.83
150	158.6 ± 11.51	7.26	1.20	178.19 ± 10.8	6.06	1.04
200	229.31 ± 16.32	7.12	1.23	263.47 ± 15.74	5.97	1.06
300	374.45 ± 22.65	6.05	1.43	430.41 ± 22.84	5.31	1.27
400	425.92 ± 26.68	6.26	1.48	567.3 ± 28.84	5.08	1.42

### 3.3 Hysteresis of dielectric layers

**FIGURE 9:**
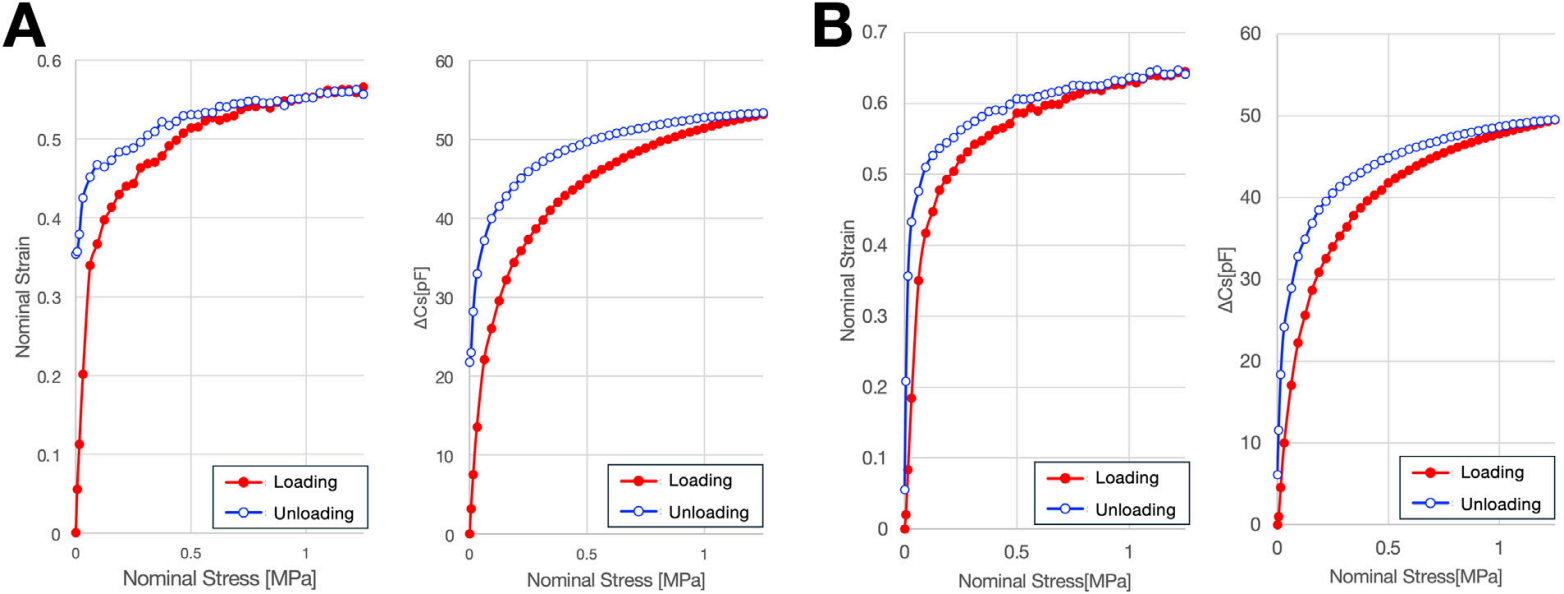
Hysteresis of materials constituting two dielectric layers. **(A)** Material constituting Dielectric Layer-1, **(B)** Material constituting Dielectric Layer-2.

For both dielectric layers, hysteresis curves were generated by plotting capacitance against nominal stress and nominal strain. These plots revealed a disparity between the loading and unloading paths, indicating the presence of hysteresis (Figure 9). The average hysteresis error with respect to both nominal stress and nominal strain was below 10% for both dielectric layer 1 and layer 2 (Table 7). Furthermore, the normalized hysteresis loop area for both layers was 0.1 or less. While this performance is not comparable to that of state-of-the-art sensors, it demonstrates a moderate level of precision.

**TABLE 7:** Differences between pressure intervals and peak capacitance intervals of force gauges before and after durability testing in slow walking simulation.

		Mean hysteresis error (%FS)	Normalized hysteresis loop area
Dielectric Layer 1	Nominal Stress	9.98	0.100
	Nominal Strain	8.13	0.075
Dielectric Layer 2	Nominal Stress	7.75	0.058
	Nominal Strain	6.28	0.050

%FS, percent of full scale.

### 3.4 Curve fitting of load-capacitance in insole-embedded sensors and verification of load estimation accuracy

The load-capacitance curve for the sensors embedded within the insole casing was regressed to a polynomial. A 5th-order polynomial, as shown Equation 9, was adopted:

$$Load\left[N\right]=a\cdot \Delta C^{5}+b\cdot \Delta C^{4}+c\cdot \Delta C^{3}+d\cdot \Delta C^{2}+e\cdot \Delta C+f$$ (9)

For example, the plot results obtained from a forefoot sensor in one casing are shown in Figure 7. From this result, the curve fitting yielded: a = 2.26 x 10^−3^, b = −9.67 x 10^−2^, c = 1.53, d = −10.4, e = 33.1, f = 1.34. The accuracy of the curve fitting was calculated as R^2^ = 0.998839 and RMSE = 3.687313 (N). We determined that the 5th-order polynomial provided a better fit for regression than a natural exponential approximation (R^2^ = 0.99856) or an integer-based exponential function (R^2^ = 0.99856), and thus adopted it.

The relationship between load and capacitance differs for each sensor. Therefore, we perform an individual calibration for each sensor embedded in the insole to determine the coefficients of Equation 9.

### 3.5 Results of dynamic characteristics analysis of insole-embedded sensors

The results for the detected pressure peak intervals are shown in Tables 8–10. No significant differences were observed in any of the conditions (p < 0.05). The signal drift rate ranged from 5% to 10%, varying with environmental and walking conditions, which suggests that correction may be necessary for long-term use (Table 11).

**TABLE 8:** Differences between pressure intervals and peak capacitance intervals of force gauges before and after durability testing in slow walking simulation.

	Temperature and humidity	Force gauge pressure interval Mean ± SD (ms)	Peak capacitance interval Mean ± SD (ms)	Difference Mean (95%CI)	
Before the Durability Test	23 °C, 50% humidity	2000.28 ± 28.24	1999.70 ± 15.49	−0.577* (−1.200, −1.061)	EQ
	40 °C, 50% humidity	2000.11 ± 31.29	1999.70 ± 21.77	−0.406* (−0.879, −0.712)	EQ
	40 °C, 80% humidity	1999.74 ± 35.65	1999.79 ± 21.28	0.047* (−0.009, 0.191)	EQ
After the Durability Test	23 °C, 50% humidity	2000.10 ± 34.71	1999.82 ± 12.35	−0.276* (−0.616, −0.466)	EQ
	40 °C, 50% humidity	2000.26 ± 34.22	1999.73 ± 18.64	−0.531* (−1.113, −0.969)	EQ
	40 °C, 80% humidity	1999.80 ± 37.28	1999.71 ± 18.69	−0.092* (−0.254, −0.105)	EQ

EQ, equivalent, * Indicates statistical equivalence, with both one-sided tests from the TOST, procedure reaching significance (p < 0.05) and the confidence interval entirely within the equivalence bounds.

**TABLE 9:** Differences between pressure intervals and peak capacitance intervals of force gauges before and after durability testing in normal walking simulation.

	Temperature and humidity	Force gauge pressure interval Mean ± SD (ms)	Peak capacitance interval Mean ± SD (ms)	Difference Mean (95%CI)	
Before the Durability Test	23 °C, 50% humidity	999.98 ± 10.08	999.96 ± 3.6	−0.021* (−0.068, −0.016)	EQ
	40 °C, 50% humidity	999.98 ± 8.76	999.91 ± 5.77	−0.064* (−0.151, −0.1)	EQ
	40 °C, 80% humidity	999.95 ± 11.18	999.81 ± 4.18	−0.140* (−0.306, −0.242)	EQ
After the Durability Test	23 °C, 50% humidity	999.86 ± 12.96	999.94 ± 4.96	0.079* (0.125, 0.184)	EQ
	40 °C, 50% humidity	1,000.04 ± 10.75	999.98 ± 4.41	−0.055* (−0.131, −0.084)	EQ
	40 °C, 80% humidity	1,000.24 ± 10.33	999.91 ± 2.89	−0.330* (−0.667, −0.625)	EQ

**TABLE 10:** Differences between pressure intervals and peak capacitance intervals of force gauges before and after durability testing in quick walking simulation.

	Temperature and humidity	Force gauge pressure interval Mean ± SD (ms)	Peak capacitance interval Mean ± SD (ms)	Difference Mean (95%CI)	
Before the Durability Test	23 °C, 50% humidity	599.94 ± 4.03	599.96 ± 3.88	0.021* (0.026, 0.058)	EQ
	40 °C, 50% humidity	599.85 ± 3.38	599.98 ± 3.45	0.128* (0.233, 0.269)	EQ
	40 °C, 80% humidity	600.02 ± 4.66	599.95 ± 5.41	−0.070* (−0.159, −0.116)	EQ
After the Durability Test	23 °C, 50% humidity	600.02 ± 4.04	600.02 ± 3.97	0.000* (−0.017, 0.017)	EQ
	40 °C, 50% humidity	600.04 ± 1.78	599.96 ± 4.75	−0.073* (−0.157, −0.13)	EQ
	40 °C, 80% humidity	600.02 ± 4.74	599.96 ± 4.94	−0.055* (−0.125, −0.09)	EQ

**TABLE 11:** Signal drift rate calculated by comparing before and after endurance testing under various temperature and humidity conditions.

Temperature and humidity environment	Slow walk	Normal walk	Quick walk
23 °C, 50% humidity	8.88%	8.21%	7.21%
40 °C, 50% humidity	8.63%	6.95%	5.81%
40 °C, 80% humidity	9.72%	8.25%	6.99%

## 4 Discussion

Polymer actuators composed of EAP and metal composites have been developed as artificial muscles because they can deform and generate stress in response to external signals (Rus and Tolley, 2015). Compared with ion-driven EAPs, electric-field-driven EAPs, including eRs, require higher driving voltages but offer faster response times and capabilities for large deformations, making them highly suitable as actuators (Bar-Cohen, 2004). Moreover, if the eR is treated as a capacitor, leveraging the instantaneous nature of the capacitance changes within the EAP, it can be applied as a sensor to inversely calculate the applied external forces. Sensing is possible with a significantly smaller voltage application–far less than the power required for actuator actuation–if minute changes in capacitance can be detected (O’Brien et al., 2010).

Integrating both sensing and actuation functionalities into an artificial muscle can not only lighten the overall system and simplify wiring but also enable the construction of real-time feedback control loops based on the state of the actuator. This mimics the reflex loops of biological proprioception, allowing adaptive movements that emulate the human body’s reflex structures (Anderson et al., 2012; Rizzello, 2023; Prechtl et al., 2024). Intrinsic Self-Sensing, which utilizes the changes in the physical properties of EAP material’s physical properties (Prechtl et al., 2024). Although this method eliminates the need for additional components, it requires the separation of the actuation signal from the sensing signal. Currently, this has not yet been achieved with the eR, posing a future challenge.

Realizing artificial muscles with self-sensing actuation capabilities is a highly challenging endeavor. In light of this, we prioritized an initial validation of their accuracy specifically as soft sensors. When using EAP-type artificial muscles solely for sensing, the most significant difference compared to conventional pressure sensors is the non-linear relationship between the external force and capacitance, primarily because of the inherent nonlinearity between stress and strain in elastomers (Wissler and Mazza, 2007). In this study, using eR, we observed an approximately linear change up to 10 N, which corresponds to a strain region of less than 10% across all conditions. However, loads beyond this range exhibit a non-linear distribution. Therefore, we measured changes in ΔC with increasing external load to investigate the eR sensor’s measurement characteristics. The coefficient of variation remained below 2% and the confidence interval width was within 3%, indicating acceptable precision and accuracy. Even when the sensor was integrated into an insole casing, although the precision slightly decreased, both the precision and accuracy were judged to be within acceptable limits. We also confirmed that a 5th-order polynomial provided the most accurate regression for the load estimation. Because the approximation formula changes with variations in the elastomer temperature, humidity, and repeated loading, we conducted measurements under multiple humidity and temperature conditions, as well as before and after 200,000 durability cycles. The results showed that while a specific relationship equation needs to be established for each sensor and temperature/humidity condition, it is entirely feasible to inversely calculate the load values based on these relationships.

**FIGURE 10:**
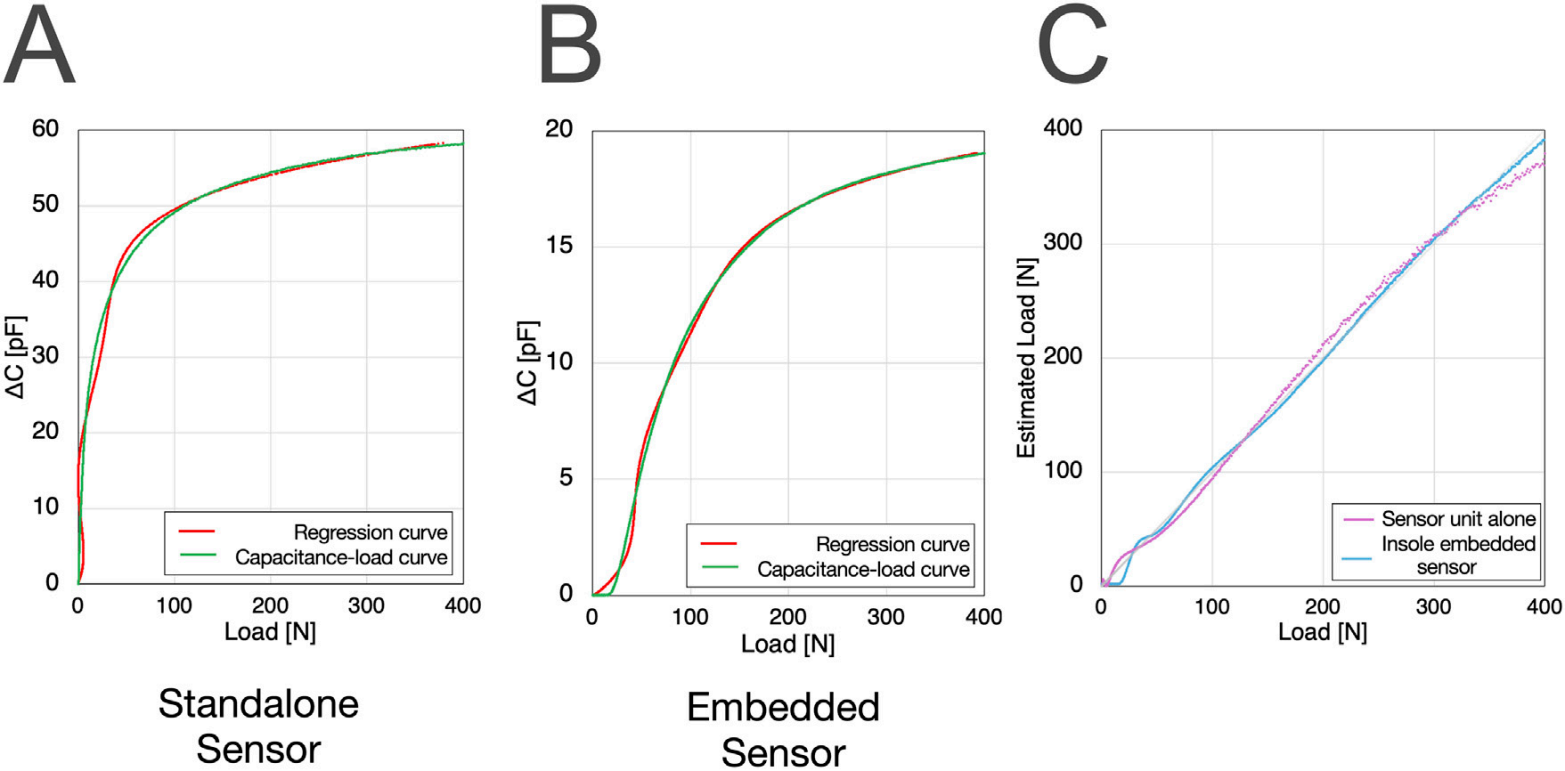
Differences in capacitance-load curve fitting between the standalone sensor and the insole-embedded sensor: The standalone sensor showed the Runge phenomenon during 5th-order polynomial regression **(A,B)**, leading to a discrepancy between actual and estimated load **(C)**.

Compared with the standalone eR sensor, the eR sensor placed within the insole casing showed a different rising profile in its load-capacitance curve owing to the gaps present within the insole casing (Figures 7, 8). A fifth-order polynomial was found to be the best fit for the load estimation from the eR sensors embedded in the insole casing, a 5th-order polynomial was found to be the best fit. For the load-capacitance curve of the standalone sensor, while the initial increase was linear, the overall non-linear behavior remained unchanged. Similarly, when using a 5th-order polynomial for regression of the standalone sensor, oscillations were observed in the regression curve and actual values below 50 N owing to the Runge phenomenon, making this regression unsuitable for that range (Figure 10). Based on these characteristics of the regression curve, the minimum detectable load was set to 50 N. When applying eR as a capacitance sensor for other purposes, where the capacitance change behavior might differ, it will be necessary to perform curve regression based on the load and capacitance changes and to determine which curve regression equation provides the best fit. Load estimation using 5th-order polynomial regression is advantageous, as it involves only a continuous process of additions and multiplications with just six parameters. This allows for real-time computation within the insole’s circuitry. Compared to machine learning-based load estimation, this method is less prone to delays during the continuous processing required for walking analysis. Furthermore, the 5th-order polynomial regression approach has an extremely small memory footprint, contributing to reduced power consumption of the smart insole.

A primary challenge in capacitive pressure sensors is managing the trade-off between high sensitivity and low hysteresis. Our design addresses this by employing a hybrid dielectric structure that pairs a soft elastomer for high sensitivity with a stiffer elastic layer to promote mechanical recovery and mitigate viscoelastic hysteresis. This multi-layer architecture not only reduces hysteresis but also extends the sensor’s dynamic range by suppressing capacitance saturation, an approach whose efficacy in improving sensor metrics is supported by the literature (Zhu et al., 2022; Kumar et al., 2023). The fabricated sensor demonstrated a hysteresis error of approximately 10% FS. While this value is higher than the sub-1% FS achieved in state-of-the-art devices (Huang et al., 2023), it is comparable to levels reported for other flexible sensors, such as the 10.3%FS error observed by Shalabi et al. (2022). Therefore, we contend that this performance represents an acceptable trade-off for applications that prioritize structural flexibility and a wide dynamic range over ultra-high precision, rendering the sensor well-suited for wearable systems aimed at the early detection of osteoarthritis by monitoring joint loading dynamics.

Over the past decade, several smart insoles have entered the market, spanning a wide price range from low to high (Table 12). This diversity is primarily due to differences in their sensor structures and intended applications. Lower-priced insoles are typically limited to specific applications, such as certain golf or running sports. Mid-to high-priced insoles, however, offer a wider range of applications, from gait analysis for research purposes to clinical use as medical devices. Common pressure sensors used in biological monitoring include piezoresistive, piezoelectric, and capacitive sensors (Hammock et al., 2013), and smart insoles utilizing the unique characteristics of each have been reported for plantar pressure measurement (Santos et al., 2024). For instance, F-Scan (Tekscan, US) uses piezoresistive sensors but is more suitable for short-term use owing to hysteresis issues (Zhang et al., 2023). In contrast, there is a growing trend towards smart insoles that adopt capacitive sensing, such as Moticon SCIENCE (Moticon ReGo, Germany) and Pedar (Novel, Germany) (Zhang et al., 2023). While capacitive sensors have drawbacks such as non-linear response and susceptibility to noise, they are suitable for long-term use owing to their low power consumption, high sensitivity, durability, and low susceptibility to hysteresis. Smart insoles using artificial muscles, such as eR, for capacitive sensing have not yet been reported. Because of the technical challenges, insoles using capacitive sensors are primarily found in the higher price range (Table 12). However, the eR Smart Insole is expected to be sold at a low to mid-price point thanks to the mass production of eR sensors and the efficiency improvements in its circuit structure.

**TABLE 12:** Summary of commercially available smart insole sensors, applications, validation, accuracy, circuit placement, and battery type.

	Product name, manufacturer	Sensors	Applications	Published accuracy/Validation	Circuit placement	Battery Type
Low price (-$300)	ORPHE TRACK ORPHE (Tokyo, Japan)	Embedded 6-axis IMU module	Running	ICC >0.9 was achieved for multiple items compared to motion capture analysis (Uno et al., 2022)	External module	Rechargeable (Built-in)
	SALTED Golf Smart Insole SALTED (Seoul, South Korea)	4-point resistive pressure sensors	Golf	Not disclosed	Integrated	Rechargeable (Built-in)
	A-RROWG NEC FiNC Technologies (Tokyo, Japan)	IMU	Walking	Not disclosed	Integrated	Rechargeable (Built-in)
	ARION Smart Insoles ATO-GEAR B.V. (Eindhoven, Netherlands)	8-point resistive pressure sensors + IMU	Running	Compared with motion capture analysis during treadmill walking, the average error of the measured items was 0.09%. (Van Hooren et al., 2023)	External module	Rechargeable (Built-in)
	NURVV Run NURVV Group (Twickenham, UK)	16-point resistive pressure sensors + IMU	Running	Not disclosed	Integrated	Rechargeable (Built-in)
Middle price ($500–2000)	Stridalyzer PRISM ReTiSense Technologies (Bangalore, India)	100-point resistive pressure matrix + IMU	Gait pressure analysis (clinical and research)	Not disclosed	Integrated	Rechargeable (Built-in)
	PRO-SPECS Smart Insole LS Networks (Seoul, South Korea)	Dual-chip IMU + pressure sensors	Step counting, posture, running data logging	Not disclosed	Integrated	Rechargeable (Built-in)
	Moticon ReGo/OpenGo Moticon ReGo AG (Munich, Germany)	16-area capacitive textile pressure sensors + IMU	Rehabilitation, sports, and gait measurement	Compared with force plate analysis, the correlation of the force-time curve was 0.8 or higher (Stöggl and Martiner, 2017)	Integrated	Replaceable Battery
High price ($4,000-)	Loadsol Novel (Munich, Germany)	Full-surface capacitive force sensor	GRF measurement, rehab, sports science	The mean bias in several items, including ground contact time, impulse, peak force, and time to peak, was <3.4%. (Seiberl et al., 2018)	External module	Replaceable Battery
	F-Scan Tekscan (Norwood,MA, United States)	954 piezoresistive sensors	Gait analysis, orthotic and footwear evaluation, and Sports biomechanics	ICC 0.83–0.98, CV 2.7%–13.4% in test–retest reliability during treadmill walking (Patrick and Donovan, 2018)	Integrated	Rechargeable (Built-in)
	Pedar novel (Munich, Germany)	99–183 Capacitive sensors	Clinical and sports gait research, footwear R&D, diabetic-foot care, rehab load monitoring, and biomechanics teaching	As a result of two measurements taken 1 week apart, 93% of the 160 parameters had a coefficient of variation of 25% or less (Ramanathan et al., 2010)	Integrated	Rechargeable (Built-in)

IMU, inertial measurement unit; GRF, ground reaction force; ICC, intraclass correlation coefficients; CV, coefficient of variation.

Accuracy validation is rarely performed for low-priced insoles, leaving their measurement precision and accuracy questionable. While most mid-to high-priced insoles undergo accuracy validation, the methodologies vary among studies. Some compare multiple gait parameters with values obtained from motion capture or force plates using intraclass correlation coefficients, while others assess the reliability of obtained values using the coefficient of variation. This diversity in validation methods makes a straightforward comparison of accuracy between different smart insoles difficult. However, our findings indicate that the measurement characteristics of the eR as an insole sensor are not inferior to those of similar capacitive sensors, demonstrating its potential for application in healthcare devices (Tao et al., 2020; Ho et al., 2022; Luna-Perejón et al., 2023).

A limitation of this study is that the load values were not continuous. Furthermore, even if the stress applied to the elastomer is constant, the capacitance fluctuates over a period, making it impossible to completely exclude hysteresis effects, where slight capacitance changes occur depending on the measurement timing. In addition, because the sensing values changed after the durability test, periodic calibration was necessary. Another limitation of this study is that our investigation was restricted to only three temperature and humidity conditions. However, because the insole inside a shoe generally operates under conditions that approximate these, despite slight variations in temperature and humidity, we believe this does not pose a significant issue for measurement. An additional limitation of this sensing approach is the increased coefficient of variation in sensor readings caused by structural gaps within the insole. As a potential solution, we considered a simplified film-type structure in which foamed urethane is sandwiched between Polyethylene Terephthalate (PET) sheets to suppress such structural variability. However, this design poses concerns regarding long-term durability under external forces. This remains a structural issue that requires further investigation in future studies. Although the eR was developed as a sensing actuator, it currently cannot perform actuation and sensing simultaneously, which requires further improvement.

In conclusion, it has been demonstrated that the eR, originally developed as an actuator, can be effectively utilized as a sensor to inversely calculate the external forces applied to the body, particularly when used as a sensor within an insole casing, based on its characteristic capacitance change. Its accuracy and precision strongly support its potential for applications beyond smart insoles in healthcare fields that require dynamic monitoring, such as gait and finger movement analysis. Furthermore, eR has the potential to be integrated as a sensor in self-sensing artificial muscles.

## Data Availability

The raw data supporting the conclusions of this article will be made available by the authors, without undue reservation.
